# Left ventricular global function index and left ventricular mass volume ratio by CMR: relation with heart failure in Thalassemia major patients

**DOI:** 10.1186/1532-429X-17-S1-P346

**Published:** 2015-02-03

**Authors:** Antonella Meloni, Petra Keilberg, Stefania Renne, Gianluca Valeri, Elisabetta Chiodi, Vincenzo Positano, Roberto Sarli, Carla Cirotto, Mari Giovanna Neri, Alessia Pepe

**Affiliations:** 1 CMR Unit, Fondazione G. Monasterio CNR-Regione Toscana, Pisa, Italy; 2 Struttura Complessa di Cardioradiologia-UTIC, P.O. "Giovanni Paolo II", Lamezia Terme, Italy; 3 Dipartimento di Radiologia, Azienda Ospedaliero-Universitaria Ospedali Riuniti "Umberto I-Lancisi-Salesi", Ancona, Italy; 4 Servizio Radiologia, Ospedaliera-Universitaria Arcispedale "S. Anna", Ferrara, Italy; 5 Centro Microcitemia, ASL Taranto Presidio Ospedaliero Orientale "M. Giannuzzi", Manduria, Italy; 6 Servizio trasfusionale, Azienda USL n° 1, Sassari, Italy

## Background

Recently two novels indicators of left ventricular (LV) performance assessed by Cardiovascular Magnetic Resonance (CMR) have been introduced: the LV global function index (LVGFI) and the LV mass/volume ratio (LVMVR). The LVGFI combines LV stroke volume, end-systolic and end diastolic volumes, as well as LV mass, integrating structural as well as mechanical behaviour. Elevated LVMVR is indicative of concentric remodelling. A LVGFI <37% and a LVMVR>1 were shown to be associated with the occurrence of cardiovascular events in no-thalassemic populations.

This retrospective cohort study aimed to systematically evaluate in a large historical cohort of thalassemia major (TM) in the CMR era whether the LVGFI and the LVMVR were associated with a higher risk of heart failure.

## Methods

We considered 812 TM patients (391 M, 30.4±8.6 years), consecutively enrolled in the Myocardial Iron Overload in Thalassemia (MIOT) network. LVGFI and LVMRI were quantitatively evaluated by SSFP cine images. The T2* value in all the 16 cardiac segments was evaluated and a global heart T2* value <20 ms was considered indicative of myocardial iron overload (MIO).

## Results

Eighty (9.9%) patients had a LVGFI<37% and, compared to the patients with a normal LVGFI, they showed a significant higher frequency of heart failure (43.8% vs 4.2%; P<0.0001). Patients with a LVGFI<37% had a significant higher risk of heart failure (odds-ratio-OR=17.59, 95%CI=9.95-21.09; P=<0.001). The risk remained significant also adjusting for the presence of MIO (OR=15.54, 95%CI=8.05-26.27; P=<0.001).

Thirty (3.7%) patients had a LVMVR≥1% and, compared to the patients with a normal LVMRI, they showed a significant higher frequency of heart failure (20.0% vs 7.7%; P=0.015). Patients with a LVMVR≥1% had a significant higher risk of heart failure (OR=3.01, 95%CI=1.18-7.64; P=0.021). The risk remained significant also adjusting for the presence of MIO (OR=3.44, 95%CI=1.31-9.01; P=0.012).

In a multivariate model including LVGFI, LVMVR and heart iron, the significant predictors of heart failure were a LVGFI<37% (OR=14.05, 95%CI=7.66-25.77; P=<0.001) and a global heart T2*<20 ms (OR=1.94, 95%CI=1.08-3.47; P=0.026).

## Conclusions

In TM patients a LVGFI<37% was associated with an higher risk of heart failure, independent by the presence of MIO. A widespread program using CMR exploiting its multi-parametric potential can have considerable power for the early identification and treatment of patients at risk for heart failure.

## Funding

The MIOT project receives "no-profit support" from industrial sponsorships (Chiesi Farmaceutici S.p.A. and ApoPharma Inc.).

